# Recrudescent Infection Supports Hendra Virus Persistence in Australian Flying-Fox Populations

**DOI:** 10.1371/journal.pone.0080430

**Published:** 2013-11-28

**Authors:** Hsiao-Hsuan Wang, Nina Y. Kung, William E. Grant, Joe C. Scanlan, Hume E. Field

**Affiliations:** 1 Department of Wildlife and Fisheries Sciences, Texas A&M University, College Station, Texas, United States of America; 2 Queensland Centre for Emerging Infectious Diseases, Department of Agriculture, Fisheries and Forestry, Archerfield BC, Queensland, Australia; 3 Department of Agriculture, Fisheries and Forestry, Toowoomba, Queensland, Australia; 4 EcoHealth Alliance, New York, New York, United States of America; Deakin University, Australia

## Abstract

Zoonoses from wildlife threaten global public health. Hendra virus is one of several zoonotic viral diseases that have recently emerged from *Pteropus* species fruit-bats (flying-foxes). Most hypotheses regarding persistence of Hendra virus within flying-fox populations emphasize horizontal transmission within local populations (colonies) via urine and other secretions, and transmission among colonies via migration. As an alternative hypothesis, we explore the role of recrudescence in persistence of Hendra virus in flying-fox populations via computer simulation using a model that integrates published information on the ecology of flying-foxes, and the ecology and epidemiology of Hendra virus. Simulated infection patterns agree with infection patterns observed in the field and suggest that Hendra virus could be maintained in an isolated flying-fox population indefinitely via periodic recrudescence in a manner indistinguishable from maintenance via periodic immigration of infected individuals. Further, post-recrudescence pulses of infectious flying-foxes provide a plausible basis for the observed seasonal clustering of equine cases. Correct understanding of the infection dynamics of Hendra virus in flying-foxes is fundamental to effectively managing risk of infection in horses and humans. Given the lack of clear empirical evidence on how the virus is maintained within populations, the role of recrudescence merits increased attention.

## Introduction

The emergence of zoonoses from wildlife represents an increasingly significant threat to global public health [1]. A number of zoonotic viral agents, including Hendra virus, have emerged from *Pteropus* species fruit-bats (commonly known as flying-foxes) since the mid-1990s [2], [3], [4], [5]. Such viruses can cause significant morbidity and mortality in humans and domestic animals [5], [6]. Hendra virus is a novel paramyxovirus, which with Nipah virus, constitutes the genus *Henipavirus*. Hendra virus has resulted in at least 75 equine deaths and four associated human deaths in eastern Australia since 1994 [3], [7], [8], [9]. All human cases have resulted from horse to human transmission.

From a human perspective, a common characteristic of many disease-causing agents is their ability to appear and disappear quickly in nature, often in an inexplicable manner. The emergence of zoonotic infectious diseases is inextricably linked to the natural system, of which the causal agent is but one part, making it extremely difficult to identify the combinations of climatic conditions, landscape structures and host characteristics that provoke a disease outbreak.

In spite of major health implications for both horses and humans, knowledge of the manner in which Hendra virus is maintained in flying-fox populations, and particularly how the virus avoids extinction as their host species become immune, remains limited [3], [10], [11]. The sporadic nature of spillover events [12], coupled with the nomadic lifestyle and complex social structure of *Pteropus* bats [13], leaves several important questions regarding the ecology and epidemiology of Hendra virus unanswered, and in particular how it persists within host populations.

Previous studies have suggested that Hendra virus is maintained through episodic infection of local populations within a metapopulation structure, but does not persist endemically within any single local population. Most hypotheses emphasize horizontal transmission within colonies via urine and other secretions, especially during pregnancy and mating [14], [15], and transmission among colonies via migration, with the magnitude of migration affected by the spatial connectivity among colonies, resulting in episodic infection [10]. While these hypotheses explain much of the observed disease dynamics, the finding of some recent virus excretion prevalence studies [16] are not adequately explained.

An alternative hypothesis, which to our knowledge has not been tested within a quantitative framework, is that endemic infection occurs via viral latency and recrudescence. Two recent studies have indicated an endemic pattern of Hendra virus infection in flying-fox populations, suggesting that the infection dynamics of this novel paramyxovirus may differ from the acute, self-limiting, episodic pattern observed with other paramyxoviruses (e.g. measles virus, phocine distemper virus, rinderpest virus) [11], [16]. In contrast to the expected episodic infection pattern, Breed et al. [11] found that the seroprevalence of Hendra virus in a spectacled flying-fox (*Pteropus conspicillatus*) population in northern Australia gradually increased over a two-year period, suggesting infection was endemic in the population over the study period. The authors found that age, pregnancy, and lactation were significant risk factors for a detectable neutralizing antibody response. Further, two recent papers suggest recrudescence as the only plausible explanation of henipavirus infection in discrete flying-fox populations. Rahman et al. reported recrudescing infection and subsequent transmission in a captive colony of *Pteropus vampyrus* in Malaysia [17], and Peel et al. reported henipavirus serologic findings in the fruit-bat *Eidolon helvum* on an isolated island in the Gulf of Ghana that also support viral recrudescence [18]. Thus, an increasing number of studies suggest another mode of henipavirus persistence may exist.

In this paper, we explore the possible role of recrudescence in the persistence of Hendra virus in flying-fox populations via computer simulation using a model that integrates published information on the ecology of flying-foxes, and the ecology and epidemiology of Hendra virus. We first describe an individual-based version of a simple SEIR (susceptible-exposed-infectious-recovered) model, modified to include the possibility that “recovered” individuals again become infectious via recrudescence during pregnancy and early lactation. We then evaluate the demographic and epidemiological dynamics generated by our new model in view of the available empirical evidence. Finally, we use our new model to explore the possible role of recrudescence in generating recurring epidemics of different magnitudes and durations in an isolated (no immigration) population under a variety of scenarios.

## Methods

### Model description

**Figure 1:**
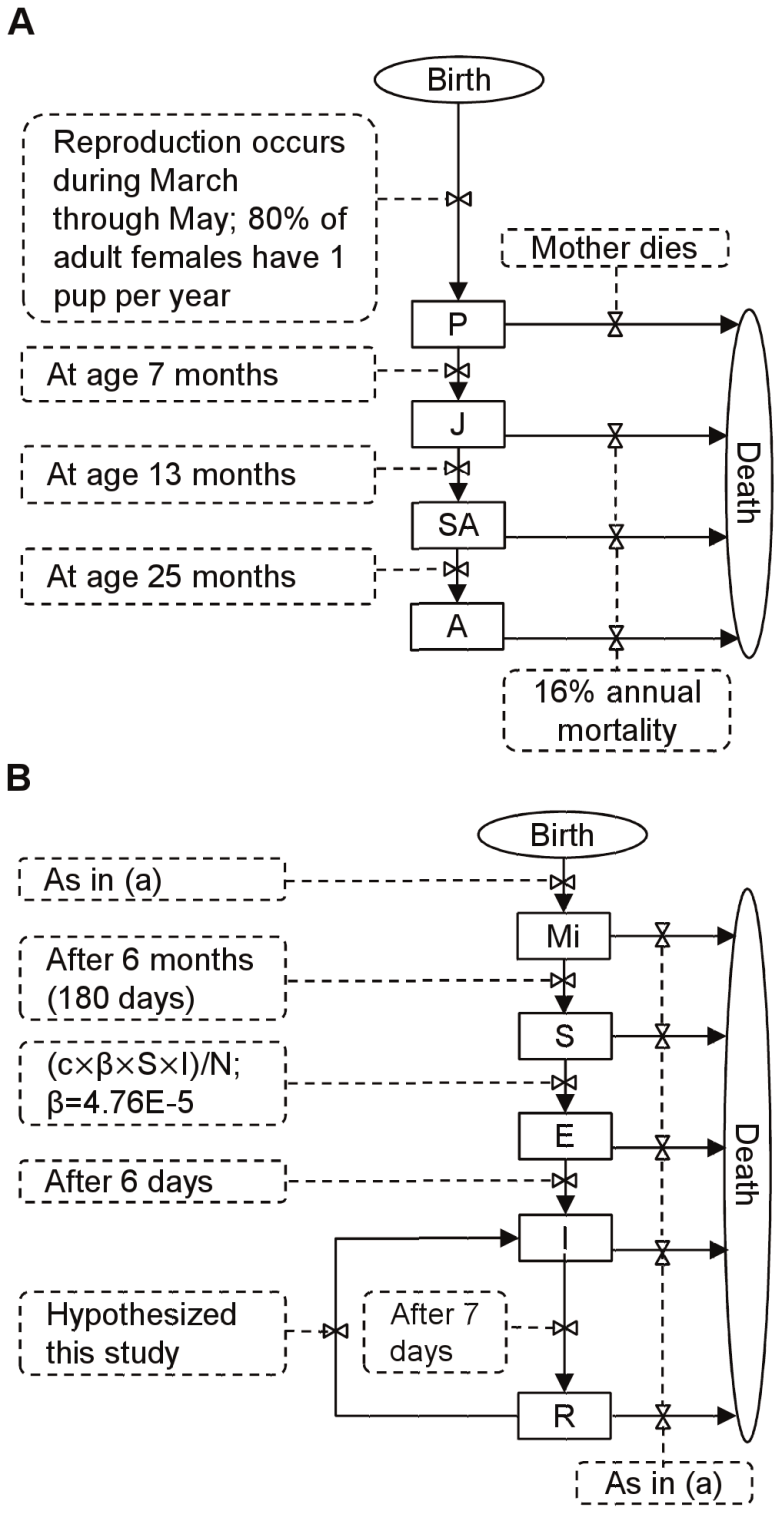
Conceptual model of the possible role of recrudescence in the persistence of Hendra virus in isolated flying-fox populations representing the daily dynamics of a colony consisting of (A) the various life history stages (pups, P; juveniles, J; pre-reproductive sub-adults, SA; reproductively mature adults, A), and (B) the various disease stages (maternally immune young-of-the-year, Mi; susceptible, S; exposed and latently infected, E; infectious, I; and recovered, R) individuals.

We formulated the model as an individual-based model representing the daily dynamics of a hypothetical black- (*Pteropus alecto*) or grey-headed (*Pteropus poliocephalus*) flying-fox population consisting of a single isolated colony. The colony is made up of male and female pups, juveniles, pre-reproductive sub-adults, and reproductively mature adults (Fig. 1A). Individuals also are classified as maternally immune (to Hendra) young-of-the-year, susceptible, exposed and latently infected, infectious, and recovered (immune but potentially recrudescent) (Fig. 1B). We assumed an initial colony size of 10,000 individuals [10] distributed among the various life history stages such as to be representative of a colony in dynamic equilibrium. That is, representative of a colony whose size varied seasonally and (stochastically) from year to year but with annual peaks remaining close to 10,000.

With regard to flying-fox life history parameters, we assumed (1) the breeding season peaks during April but extends into March and May, (2) each year, on average, 80% of the mature females have one pup, (3) gestation lasts 26 weeks, (4) the majority (≈ 90%) of births occur during October with the remainder occurring in September and November, and (5) the period of pup dependency on their mothers lasts 5 months [19], [20], [21]. We classified individuals aged 0 to 6 months as pups, 7 to 12 months as juveniles, 13 to 24 months as sub-adults, and older than 24 months as sexually mature adults [10]. We assumed an age-independent annual mortality rate of 16% [22], except for pups; we assumed pups survive unless their mother dies.

With regard to epidemiological parameters of Hendra virus, we assumed (1) a transmission rate of 0.0000476, (2) a viral latency (incubation) period lasting 6 days after infection, followed by (3) an infectious period lasting 7 days [10], as well as (4) a period of immunity for pups birthed by infectious or recovered mothers lasting from 4 to 6 months, with most pups losing maternal immunity by 5 months of age [11], [15]. We calibrated the contact rate such that a population with 95% susceptible individuals at the beginning of an epidemic (at the appearance of the first infectious individual) would attain a maximum of 60% infectious individuals during the epidemic [14]. We assumed: (1) recrudescence occurs stochastically in females during pregnancy and early lactation [11]; (2) the individual becomes infectious immediately upon recrudescence (i.e., there is no latency period); and (3) the same individual can recrudesce only once within any given reproductive period. We modelled different rates of recrudescence consistent with current hypotheses in the scientific literature [11], [17], [18].

We have provided a detailed description of the model, following the ODD (Overview, Design concepts, Detail) protocol [23] for individual-based models in Appendix S1. We programmed the model in NetLogo [24].

### Model evaluation

To evaluate the usefulness of the model in exploring the possible role of recrudescence in the persistence of Hendra virus, we confirmed the ability of the model to simulate adequately the basic life cycle pattern of individuals (breeding, pregnancy, birth, lactation, and pup dependency), the resulting seasonal patterns, the stage-class structure of the population (pups, juveniles, sub-adults, adults), and the stable age-class distribution, implicitly under constant environmental conditions which would result in long-term stability in population size. We imposed the basic life cycle pattern via fixed rules coded into the model, and seasonal patterns in age structure shifts appeared as a direct result of individual life cycles. The ability of the model to generate appropriate demographic patterns was a prerequisite for the subsequent representation and explanation of disease dynamics. For this phase of model evaluation we ran three Monte Carlo simulations using baseline parameter values (see model description in Appendix S1).

We then confirmed the ability of the model to generate differences in the magnitudes and durations of epidemics as a function of the proportions of susceptible individuals in the population at the beginning of the epidemic. Once again, although we imposed these patterns via fixed rules coded into the model, the ability of the model to generate reasonable patterns of local epidemics, from initial infection to apparent viral extinction, was a prerequisite for the subsequent evaluation of the role of recrudescence in disease persistence. Thus, for simulations during this phase of model evaluation, we assumed no recrudescence and initiated epidemics by introducing one infectious individual into the population on day 1 of simulated time (1 January), while varying the initial proportions of susceptible individuals in the population from 0.95 to 0.05 in increments of 0.05. We ran three Monte Carlo simulations for each initial proportion of recovered individuals, with all other parameters set to their baseline values, and monitored the (1) magnitudes of epidemics (maximum proportion of infectious individuals in the population), (2) numbers of days post infection that these maximums occurred, and (3) durations of epidemics (number of days with the proportion of infectious individuals >0.10).

Finally, we evaluated the relative sensitivity of the epidemiological dynamics of our model to changes in the values of important parameters, and the robustness of these responses to the manner in which epidemics were initiated (via recrudescence or immigration). Sensitivity analysis [25] focused on determining the response of the magnitude and duration of epidemics to variation in (1) initial population size, (2) initial proportion of recovered individuals in the population, (3) duration of latency period, (4) duration of infectious period, (5) duration of maternal immunity period, (6) disease transmission rate, and (7) recrudescence rate. Except for disease transmission rate and recrudescence rate, we selected the range of values used for the various parameters (Table 1) to correspond to the ranges reported in [10] (Table 1). We varied disease transmission rate between 2.0E-5 (the lower end of the range reported in [10]) and 0.001, and we varied recrudescence rate over this same range. Robustness analysis consisted of comparing the relative sensitivities of two versions of the model to changes in the values of these parameters. The first version assumed epidemics were initiated by recrudescence and the second assumed epidemics were initiated by introducing one infectious immigrant into the population on a randomly-chosen day of the year (assuming no recrudescence). For each sensitivity analysis, we once again ran three Monte Carlo simulations for each parameter combination.

**Table 1:** Experimental design for the sensitivity analysis and robustness analysis.

Parameter	Minimum	Mid-range	Maximum
Initial population size	5000	10000	15000
Initial proportion of R individuals	0.05	0.5	0.95
Duration of latency period (days)	4	6	9
Duration of infectious period (days)	4	7	10
Duration of maternal immunity period (days)	150	180	210
Disease transmission rate	2.0E-5	1.0E-4	0.001
Recrudescence rate (sensitivity analysis)	2.0E-5	1.0E-4	0.001
Recrudescence rate (robustness analysis)	0	0	0

Three Monte Carlo simulations were run for each parameter combination.

### Role of recrudescence in the persistence of Hendra virus

After confirming the ability of our model to produce and explain differences in the magnitudes and durations of Hendra epidemics based on the life cycle of flying-foxes and the epidemiology of Hendra virus in a manner consistent with current knowledge, we used the model to explore the possible role of recrudescence in generating recurring epidemics in an isolated population under a variety of scenarios. In one series of simulations, we varied recrudescent rates from one simulation to the next with the rate set at either 1.0E-5 or 5.0E-5 or 0.001. In a second series, we varied the frequency of occurrence of recrudescence such that a year with a high recrudescence rate (0.001) was followed by from 1 to 19 consecutive years with no recrudescence. For each series of simulations, we once again ran three Monte Carlo simulations for each scenario and recorded the magnitudes and durations of the resulting epidemics. We set all parameters other than those controlling recrudescence to their baseline values, and initialized each of these simulations with 95% of the population consisting of susceptible individuals.

## Results

### Model evaluation results

**Figure 2:**
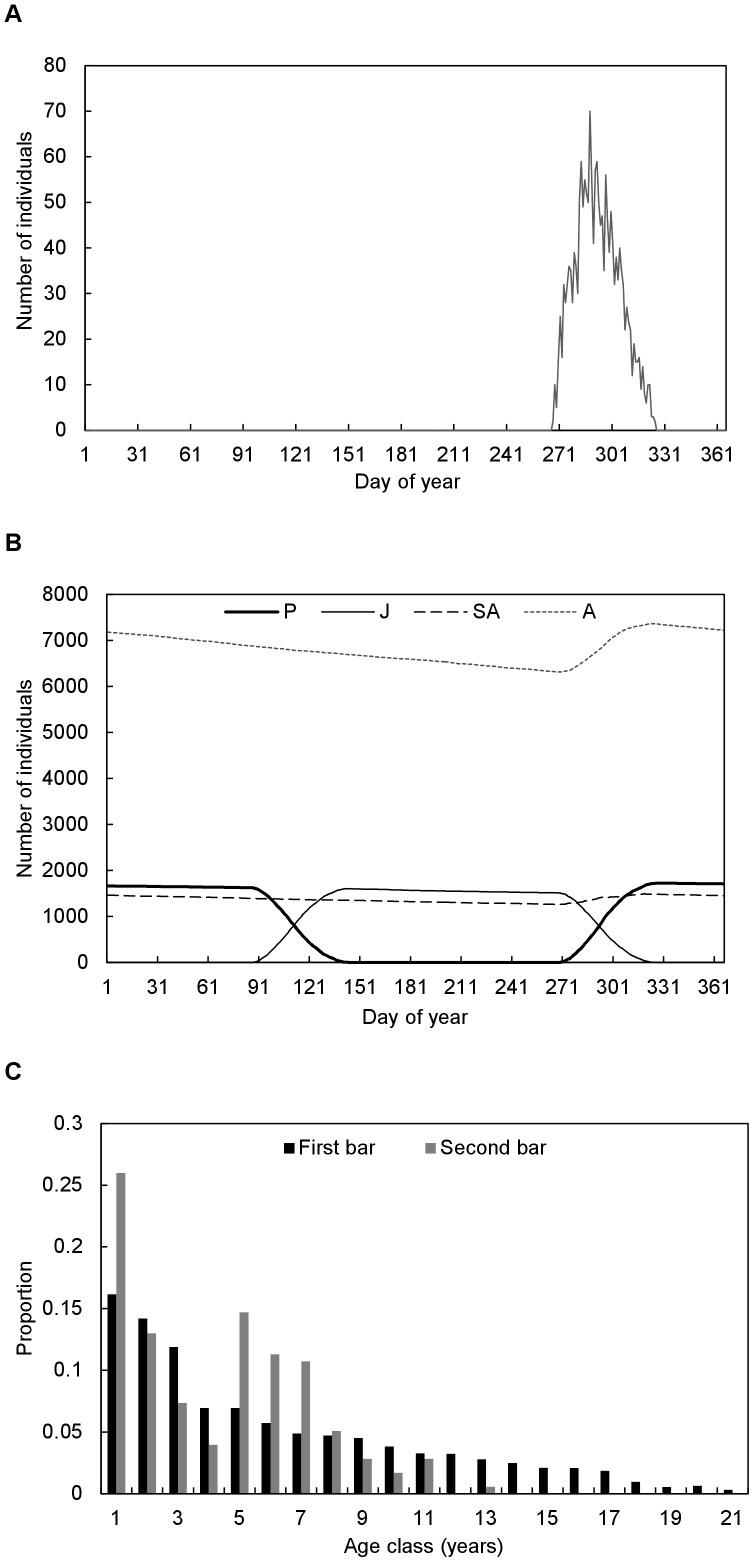
The (A) seasonality of births, (B) seasonal fluctuations in stage-class structure, and (C) stable age-class distribution of the simulated population (first bar), implicitly under constant environmental conditions which would result in long-term stability in population size. Also shown in (C, second bar) is the age-class distribution reported in [29].

The basic life cycle pattern of breeding, pregnancy, and the resulting births (Fig. 2A), as well as the seasonal fluctuations in stage-class structure (Fig. 2B) were similar to those reported in field studies [14], [26], [27], [28]. The simulated stable age-class distribution (Fig. 2C) was qualitatively similar to that observed in [29], with differences most likely resulting from the age-specificity and year-to-year variability of mortality rates in the field, and sizes of the simulated populations remaining relatively stable.

**Figure 3:**
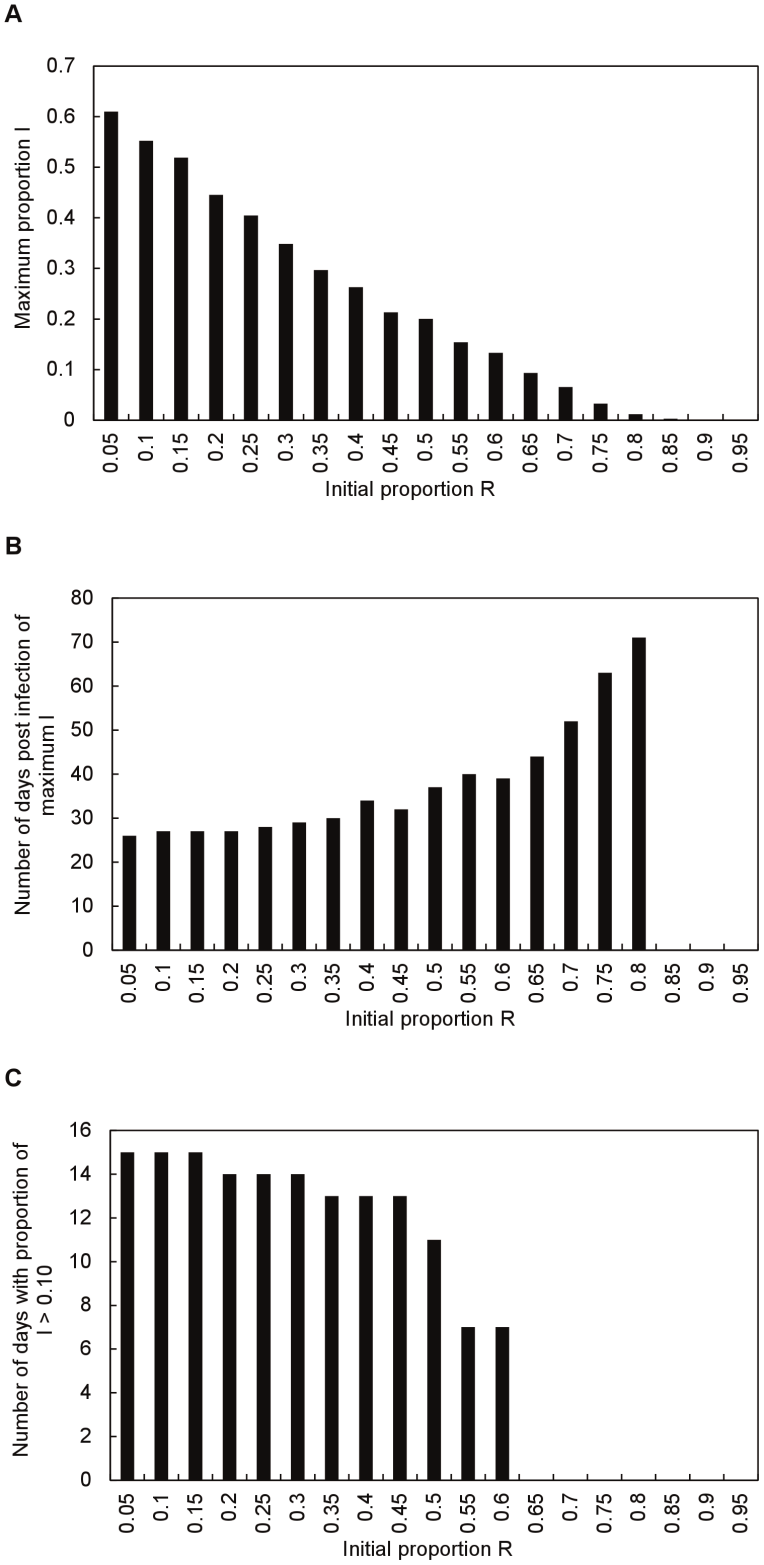
Simulated differences in (A) magnitudes of epidemics (maximum proportion of I individuals in the population), (B) time lag between initial and peak infection, and (C) durations of epidemics (number of days with proportion of I>0.10) as a function of the initial proportion of R individuals in the population at the beginning of the epidemic.

Magnitudes and durations of simulated epidemics were consistent with observations that the primary factor limiting infections was the number of susceptible individuals rather than the number of infectious individuals [30], [31], and were qualitatively similar to the dynamics of local epidemics produced by the model in [10]. That is, depletion of the pool of susceptible individuals led to local viral extinction, with the magnitudes of epidemics decreasing (Fig. 3A), the time lag between initial infection and peak infection increasing (Fig. 3B), and the durations of epidemics decreasing (Fig. 3C) with increasing proportions of recovered (hence, decreasing proportions of susceptible) individuals in the population at the time of initial infection. The model in [10] suggested that the threshold proportion of susceptible individuals required for disease invasion into their simulated population was approximately 0.3. Our simulations suggested that the pathogen could not sustain itself within the host population if the proportion of susceptible individuals was less than approximately 0.2 (proportion of recovered individuals >0.8) at the time of invasion (Fig. 3A), that the time from initial infection to peak infection increased exponentially as the proportion of susceptible individuals at the time of invasion approached this threshold (Fig. 3B), and that the proportion of infectious individuals within the population would not reach 0.1 unless the proportion of susceptible individuals at the time of invasion exceeded approximately 0.4 (proportion of recovered individuals <0.6) (Fig. 3C). Once again, although these behaviours are fundamental consequences of the SEIR-model paradigm dynamics, which we imposed via fixed rules coded into the model, confirmation of the ability of the model to generate these patterns was a prerequisite for subsequent applications of the model.

**Figure 4:**
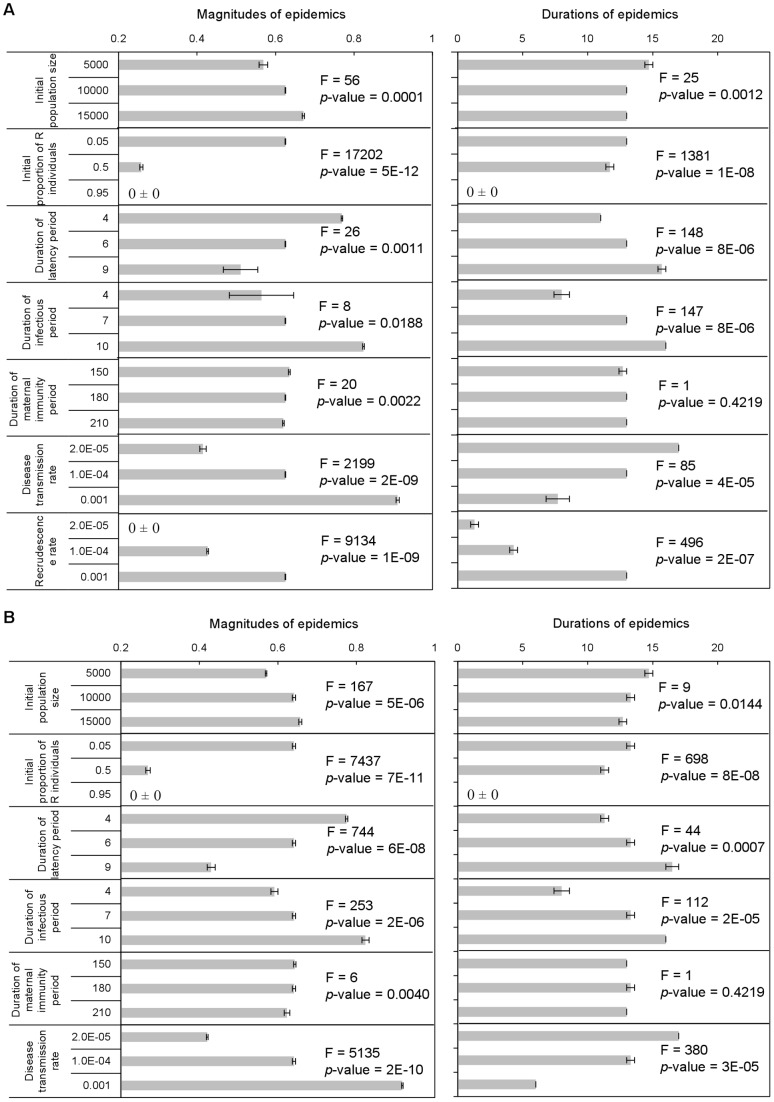
Simulated differences in the magnitudes of epidemics (maximum proportion of I individuals in the population) and durations of epidemics (number of days with proportion of I>0.10) resulting from changes in the values of important model parameters in versions of the model assuming epidemics were initiated (A) via recrudescence and (B) via immigration (Error bars represent ± SE).

Results of sensitivity analyses indicated that both magnitude and duration of epidemics were most sensitive to variation in the proportion of recovered individuals in the population at the initiation of the epidemic, regardless of the manner in which epidemics were initiated (via recrudescence or via immigration) (Fig. 4). These results again were consistent with the general consensus that availability of susceptible individuals is the primary infection-limiting factor [30], [31], and also were comparable to the simulation results in [10]. As expected, magnitudes and durations of epidemics produced by both versions of the model also were sensitive to changes in disease transmission rate and the lengths of the latency and infectious periods (Fig. 4). There were no statistically significant differences between the two versions of the model with regard to their sensitivity to changes in any of the parameters tested (Tables 2 and 3). Thus, relative sensitivity of simulated local (within a single population) epidemiological dynamics to changes in the values of important parameters was consistent with current knowledge, and these dynamics were robust to the manner in which epidemics were initiated (via recrudescence or immigration).

**Table 2:** Results (F ratios/*p*-values) of two-way ANOVAs comparing relative sensitivities of magnitudes of epidemics produced by versions of the model assuming epidemics were initiated via recrudescence or via immigration.

		Epidemics initiated via recrudescence					
		Initial population size	Initial proportion of R individuals	Duration of latency period	Duration of infectious period	Duration of maternal immunity period	Disease transmission rate
Epidemics initiated via immigration	Initial population size	0.0506 (0.8257)	**−−**	**−−**	**−−**	**−−**	**−−**
	Initial proportion of R individuals	**−−**	4.4751 (0.0626)	**−−**	**−−**	**−−**	**−−**
	Duration of latency period	**−−**	**−−**	1.7320 (0.2127)	**−−**	**−−**	**−−**
	Duration of infectious period	**−−**	**−−**	**−−**	0.2599 (0.6194)	**−−**	**−−**
	Duration of maternal immunity period	**−−**	**−−**	**−−**	**−−**	2.7631 (0.2123)	**−−**
	Disease transmission rate	**−−**	**−−**	**−−**	**−−**	**−−**	2.6743 (0.3675)

**Table 3:** Results (F ratios/*p*-values) of two-way ANOVAs comparing relative sensitivities of durations of epidemics produced by versions of the model assuming epidemics were initiated via recrudescence or via immigration.

		Epidemics initiated via recrudescence					
		Initial population size	Initial proportion of R individuals	Duration of latency period	Duration of infectious period	Duration of maternal immunity period	Disease transmission rate
Epidemics initiated via immigration	Initial population size	2E-14 (1.0000)	**−−**	**−−**	**−−**	**−−**	**−−**
	Initial proportion of R individuals	**−−**	0.0000 (1.0000)	**−−**	**−−**	**−−**	**−−**
	Duration of latency period	**−−**	**−−**	1.5000 (0.2442)	**−−**	**−−**	**−−**
	Duration of infectious period	**−−**	**−−**	**−−**	0.1429 (0.7121)	**−−**	**−−**
	Duration of maternal immunity period	**−−**	**−−**	**−−**	**−−**	2.0000 (0.1827)	**−−**
	Disease transmission rate	**−−**	**−−**	**−−**	**−−**	**−−**	2.0000 (0.1827)

### Role of recrudescence

**Figure 5:**
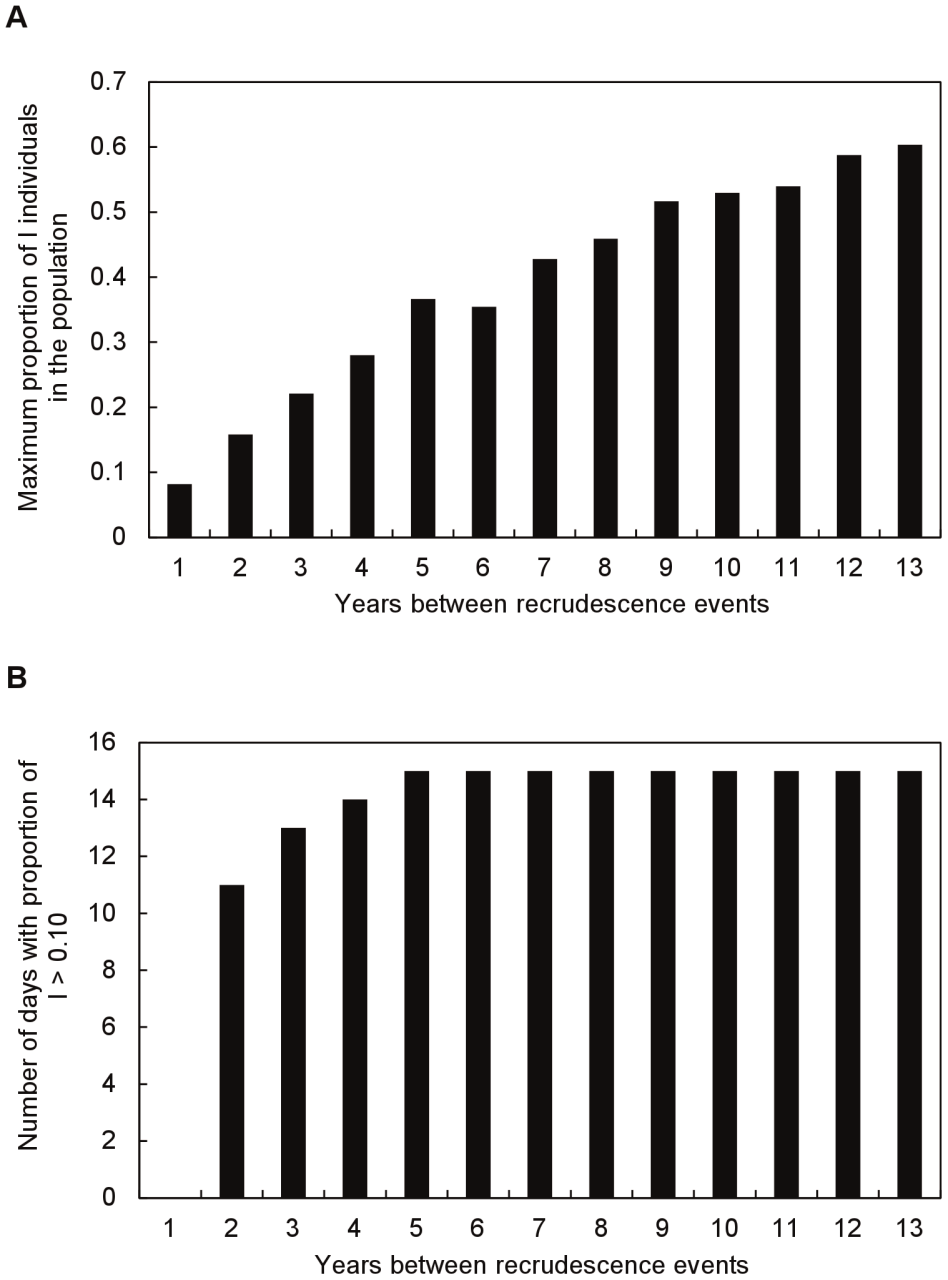
Simulated differences in the (A) magnitudes of epidemics (maximum proportion of I individuals in the population) and (B) durations of epidemics (number of days with proportion of I>0.10) resulting from the indicated number of consecutive years without recrudescence between years with a high recrudescence rate.

Results of simulations exploring the role of recrudescence suggested that magnitudes and durations of initial and subsequent epidemics remained essentially the same as recrudescent rates were increased from 5.0E-5 to 0.001, with these rates producing an initial epidemic that infected about 60% of the population with subsequent annual epidemics infecting about 2% of the population. During the initial epidemics, infection levels remained above 10% for about 2 weeks, whereas during subsequent epidemics infection levels remained above 2% for about 3 weeks. With a recrudescent rate of 1.0E-5, initial epidemics also infected about 60% of the population and remained above 10% for about 2 weeks. However, the initial epidemic could occur in any of the first 4 or 5 years, or not occur at all during a 20-year simulation, and was never followed by subsequent epidemics. Results of simulations in which we increased the number of years between recrudescence events suggested the magnitudes of epidemics increased at a decreasing rate from slightly less than 10% to about 60% of the population infected as the number of years between events increased from one to 13, and remained at about 60% as the number of years between events increased from 13 to 20 (Fig. 5A). The time that infection levels remained above 10% increased at a decreasing rate from 11 to 15 days as the number of years between events increased from two to five, and remained at 15 as the number of years between events increased from five to 20 (Fig. 5B).

## Discussion

The work reported here shows that recrudescence provides an explanation for the persistence of Hendra virus infections in an isolated flying-fox population (as indicated in [11], [16]). Previously, metapopulation models of the presence of Hendra virus in flying fox populations had provided the most coherent explanation of the patterns of infections observed [10], [32]. We do not suggest that recrudescence is more important than transmission via immigration in initiating local epidemics or that the two mechanisms are mutually exclusive. We suggest that recrudescence provides a plausible mechanism of Hendra virus persistence in flying-fox populations, and in addition, a parsimonious model for maintenance of Hendra virus in populations that are ecologically isolated.

Hendra virus could be maintained in an isolated flying-fox population indefinitely via periodic recrudescence in a manner such as to be indistinguishable from maintenance via periodic immigration of infected individuals. The magnitude and duration of simulated epidemics initiated by either mechanism of infection depended on the proportion of susceptible individuals in the population at initiation of the epidemic. This is consistent with theory [33] and has been reported in earlier field studies of flying-foxes [10], [14] and in pigs with another henipavirus, Nipah virus [34]. The magnitudes of our simulated epidemics appeared to increase almost linearly with the length of time between recrudescent events. The variability in magnitudes of epidemics after any given recovery period was accounted for primarily by annual variation in cohort sizes (numbers of pups born) and hence, annual variation (lagged one year) in recruitment of susceptible individuals into the population. However, after relatively long periods with no new infections in the population, the dwindling number of potentially recrudescent individuals became the factor limiting the initiation of an epidemic.

The usefulness of our model and its results firstly rests on the credibility of the model, and secondly on how well it explains observed events in nature. To evaluate model credibility, we followed the Pattern-Oriented Modelling protocol as outlined in [35], which focuses on comparing patterns in the structure and dynamics of the model with those patterns in the real system deemed most relevant to the questions of interest. The general idea is that the credibility/usefulness of a model is determined not only by its ability to generate a single pattern of interest, but also by its structural realism. This requires that the model produces multiple independent patterns that match observations from the real system and that collectively explain how the pattern of interest was generated [36]. We assessed the model to be credible/useful based on its ability (1) to generate demographic patterns representing appropriate seasonality of births and fluctuations in population size, as well as appropriate lengths of developmental stages and stage-class composition of the population; (2) to generate epidemiological patterns representing magnitudes and durations of epidemics that demonstrated appropriate sensitivity to changes in the values of important parameters affecting disease transmission (such as transmission rate, lengths of periods of latency, infectiousness, and maternal immunity, and initial proportion of immune individuals in the population); and (3) to generate these epidemiological patterns robustly, that is, regardless of the mechanism, recrudescence or immigration, used to initiate epidemics.

The robustness of simulation results to changes in the process used to initiate epidemics, together with the empirical evidence for the recrudescence of Hendra virus infections in mammalian hosts provides evidence for the potential role of recrudescence in the persistence of Hendra virus in flying-fox populations. We suggest that recrudescence is a possible mechanism for initiating local epidemics; we do not suggest that recrudescence is more, or less, important than transmission by immigration, nor that the two mechanisms are mutually exclusive. Plowright et al [10], [15] argue that the problematic aspects of persistence via migration and horizontal transmission diminish under several hypothesized scenarios. For example, if some young-of-the-year receive maternal immunity (which subsequently wanes), the period of recruitment of susceptible individuals to the population would be extended [15]. Further, if anthropogenic perturbations have altered the spatial structure of the metapopulation, this may result in changed spatial dynamics and connectivity between colonies [10]. However, given the lack of clear empirical evidence indicating the manner in which Hendra virus is maintained within populations, we do suggest that the recrudescence concept remains plausible and merits increased attention. We also would note that our simulated patterns of infection agree with the infection patterns observed in the field in [11], and that our model structure per se provides a set of ecologically/epidemiologically interpretable cause-effect relationships capable of generating those patterns. Breed et al. [11] found increased levels of seroprevalence of HeV during late pregnancy and the first few weeks of lactation, suggesting an endemic infection with periodic pulses of viral transmission during pregnancy. These authors further interpreted their findings as being consistent with a scenario of persistent infection with viral latency and recrudescence, as had been proposed by H.E. Field (unpublished data). Breed et al. [11] also noted that an increase in viral transmission associated with pregnancy is consistent with the temporal pattern of some Hendra virus spillover events to horses. The observed temporal synchrony of spill-over events from flying-fox populations separated from each other by considerable distances also raises some questions concerning the ability of the immigration of infected individuals to generate such synchrony.

In contrast to the expected episodic infection pattern, Breed et al. [11] observed that seroprevalence of Hendra virus in a spectacled flying-fox population in northern Australia gradually increased over a two-year period, suggesting infection was endemic in the population over the study period. They found that pregnancy and lactation were significant risk factors for the presence of neutralizing antibodies to Hendra virus, with antibody titres being significantly higher in females than males, and with the highest titres occurring in pregnant animals. They also found temporal variation in antibody titres, suggesting that immunity to the virus may wax and wane on a seasonal basis. Plowright et al. [15] also identified a serological association with age, pregnancy and lactation, and a positive Hendra virus serostatus. In our model, we discounted the significance of lactation because these previous studies analysed serological data not infection data. Given the described association between pregnancy and a positive serostatus, the association between lactation and serostatus likely reflects the same infection event, putatively in (the preceding) pregnancy. Similarly, we discounted a specific association between late pregnancy and a positive serostatus [11]. Those authors did not suggest a differential association between late versus early pregnancy and serostatus, but rather that the association resulted from the ability to accurately detect late pregnancy (but not early pregnancy) with the technique used.

Recrudescing henipavirus infections have been reported in humans, although infectiousness following recrudescence has not been described. Field and Kung [12] noted that henipavirus infection in humans has been associated with viral latency and subsequent recrudescence. In addition, apparent recrudescence has been reported in flying-foxes, with evident bat-to-bat transmission following recrudescence [11]. These findings suggest infectiousness following recrudescence in flying-foxes, but not in humans, a scenario not unexpected given the likely different pathogenesis and immune dynamics in the natural host and in spillover hosts. Persistence of other viral diseases such as measles has been associated with population heterogeneity (age structure, social structure) and population spatial structure (metapopulations existing in spatially discrete patches) [37], [38]. Population heterogeneity facilitates the persistence of infection if different population segments are infected at different times. Population spatial structure facilitates the persistence of infection if local infections in spatially-discrete patches die out sequentially rather than simultaneously and allow reinfections to occur through migration between patches.

In addition to recrudescence providing a plausible mechanism of Hendra virus persistence in flying-fox populations, it also plausibly explains the often observed ‘pattern’ of equine cases, namely temporal clustering over spatially disparate locations. While this pattern is likely associated with a suite of ecological, climatic and other factors, the presence of virus obviously represents the ‘necessary’ component within an epidemiological ‘necessary and sufficient’ causality paradigm. Twenty-eight of the 42 incidents of Hendra virus infection in horses reported to date have occurred in June (8), July (13), or August (7) [39]. Our results suggest that recrudescence in a single female sometime during pregnancy would be sufficient to precipitate a pulse of infection in the preceding year's pups (≈10% of the population) and other susceptible individuals in the colony, with the peak infection prevalence occurring during one of these months, even if an epidemic had passed through the population the previous year. Were this scenario to occur in disparate colonies, it would facilitate disparate equine cases clustered in time. Thus, to the extent that risk of infection in horses is related to viral prevalence in flying-foxes, post-recrudescence pulses of infectious flying-foxes provide a plausible causal mechanism for the observed pattern of infection in horses.

Clearly, an accurate understanding of the infection dynamics of Hendra virus in flying-fox populations is crucial for the development of effective management strategies to reduce the risk of disease spillover from flying-foxes to horses and humans. Although simulated disease dynamics produced by immigration of infected individuals versus recrudescence were indistinguishable when viewed from the perspective of a local colony, which was the focus of the present study, our results suggest that differences should emerge when viewed from a meta-population perspective. Simulation of the effect of connectivity (level of immigration) among colonies on meta-population disease dynamics in flying foxes (assuming no recrudescence) indicated that median frequencies and sizes (maximum amplitudes) of local epidemics were related directly and inversely, respectively, to level of connectivity [10]. These results can be predicted theoretically based on the epidemiological concept of a threshold susceptible population size [40] together with the often-assumed analogy between aggregations of hosts and pathogen habitat patches within a meta-population dynamics context [41]. Pathogen dynamics depend on the level of mixing within local host populations (number and frequency of contacts between infected and susceptible individuals), as well as the level of connectivity among host sub-populations (number and frequency of immigrants from infected to susceptible sub-populations). Based on our results, if a significant proportion of local epidemics is initiated by recrudescence of resident individuals, then we would not expect to find a significant relationship between median epidemic frequencies or sizes and level of connectivity among colonies.

Additionally, field observations may facilitate hypothesis-testing. If recrudescence of resident individuals is a significant source of new infections, we would expect that the variability in proportion of infected individuals within regions and among regions would not differ significantly. Hopefully, our ideas will promote increased investigation into the importance of recrudescence as a mechanism contributing to the persistence of Hendra virus in flying-fox populations.

## Supporting Information

Appendix S1 **Detailed model description.**
(DOCX)
