# Supplementary material for: Recrudescent Infection Supports Hendra Virus Persistence in Australian Flying-Fox Populations
Source: PLoS One. 2013 Nov 28;8(11):e80430. doi: 10.1371/journal.pone.0080430 (PMC3842926; doi:10.1371/journal.pone.0080430)
Supplement: Appendix S1 — Detailed model description. (DOCX) [file pone.0080430.s001.docx]

**Appendix S1**

**Detailed model description**

Detailed model description following the ODD (Overview, Design concepts, Detail) protocol for individual-based models developed in [1].

***Overview.*** (1) Purpose: The purpose of the model is to explore the possible role of recrudescence in the persistence of Hendra virus within isolated flying-fox populations.

(2) Entities, state variables, and scales: Model entities include flying-foxes. Flying-foxes are characterized by state variables representing (a) the ID# of their mother, (b) sex, (c) age (days), (d) stage (pup, P; juvenile, J; sub-adult, SA; adult, A), (e) infection status (susceptible, S; exposed and latently infected, E; infectious, I; recovered, R; maternally immune, Mi), (f) latency counter (count of days from beginning to end of latency period for E individuals; always 99999 for S, I, R, and Mi individuals), (g) infectious counter (count of days from beginning to end of infectious period for I individuals; always 99999 for S, E, R, and Mi individuals), (h) pregnancy counter (count of days from conception to birth; always 99999 for males and non-pregnant females), and (i) pup dependency counter (count of days from birth to end of period of pup dependency on mother; always 99999 for males and females without a pup). The time step is one day and simulations are run for periods up to 20 years. The spatial extent of the modelled system is the area encompassing the normal activity ranges of the flying-foxes that form a single colony.

(3) Process overview and scheduling: The following processes are executed in the given order each time step. During each process, the order in which individual flying-foxes act (i.e., are given the opportunity to die, recrudesce, breed, etc.) always is randomized. State variables representing the ages and stages of flying-foxes are updated at the beginning of each time step. All other state variables are updated immediately after an action affecting the state variable is performed: (a) General state of system updated. (b) Deaths occur. (c) Recrudescence occurs. (d) Breeding occurs. (e) Births occur. (f) Disease transmission occurs. (g) System-level output produced.

***Design concepts.*** (1) Basic principles: The basic principle underlying the model’s design is that viral latency and subsequent recrudescence can play an important role in the persistence of viral diseases within populations. An apparent recrudescing infection and subsequent transmission of Nipah virus (related to Hendra virus) has been reported [2], and the question of a plausible role for recrudescence in Hendra virus persistence at the population level has been raised [3]. The latency/recrudescence hypothesis provides an alternative to the currently-favoured metapopulation model, which requires sequential “re-seeding” of the infection via immigration in sub-populations to sustain the infection within the metapopulation. The model is designed to represent a stable (births approximately equal to deaths; birth and death are stochastic processes), isolated (no immigration or emigration) population in which individuals that have recovered from the disease can again become infectious with specified probabilities, the values of which can be varied from simulation to simulation and/or during simulations.

(2) Emergence: The most important outputs of the model are the number and proportion of infectious individuals in the population over time. These outputs emerge at the population level from the interactions of individuals, which are imposed by fixed rules coded into the model.

(3) Adaptation, Objectives, Learning, Prediction: Agents (flying-foxes) do not explicitly adapt to their environment, seek to achieve specific objectives, learn from past actions, or predict the outcomes of their actions.

(4) Sensing: Flying-foxes are implicitly aware of their own condition (sex, age, stage, infection status, latency status, infectious status, pregnancy status, pup dependency status, maternal immunity status, reproductive stress level), and also the infection status of all of the other flying-foxes in the system.

(5) Interaction: Flying-foxes interact directly via disease transmission (infectious individuals cause susceptible individuals to become infectious). Flying-foxes implicitly interact each day with all other flying-foxes within the area encompassed by the normal activity ranges of the flying-foxes that form a single colony. Grey-headed flying foxes may show cumulative displacement distances of 260 km over a 10-week period [4].

(6) Stochasticity: Initial sex, age, and infection status are assigned to individuals stochastically. Deaths, births, and disease transmission at the individual level are represented as stochastic processes based on empirically-determined probabilities to generate variability in simulated population-level output that can be compared with the variability in population-level empirical data. A more detailed representation of the specific mechanisms responsible for the variability in these processes is unwarranted. Recrudescence also is represented as a stochastic process based on hypothetical probabilities that are consistent with current hypotheses in the scientific literature.

(7) Collectives: Collectives in the model include the number of S, E, I, and R individuals in the population, and the total number of individuals in the population, which together affect the probability of disease transmission among individuals.

(8) Observation: The model monitors the daily numbers and proportions of flying-foxes in each stage and infection status, with particular interest in temporal dynamics of number and proportion of I individuals in the population related to the timing and rate of recrudescence.

***Details.*** (1) Initialization: The model is initialized on January 1 with 10,000 flying-foxes approximately equally divided between males and females, with the ages of individuals distributed such as to approximate the stable age-class distribution. The distribution of individuals among S, E, I, and R individuals depends upon the scenario being simulated. All counters representing latency, infectious, pregnancy, pup dependency, and maternal immunity statuses are initialized.

(2) Input data: No input data are required.

(3) Sub-models: (a) General state of system is updated. Calendar date is advanced one day. Ages of flying-foxes are increased by one day. Stages of flying-foxes are updated, as appropriate: pups become juveniles at 6 months of age, juveniles become sub-adults at 1 year of age, sub-adults become adults at 24 months of age, which is the age at which they reach sexual maturity [5]. (b) Deaths occur stochastically for juveniles, sub-adults, and adults based on a fixed daily probability which represents an estimated annual death rate of 16% [6]. Pups do not die unless their mother dies, in which case they die immediately. (c) Recrudescence occurs stochastically in females during the second two-thirds of their pregnancy and during the first two months of lactation at a variety of rates, depending on the hypothesis being investigated. The timing and probabilities of occurrence are hypothetical, but are consistent with current hypotheses in the scientific literature [7]. Once recrudescence occurs, the individual becomes infectious immediately and remains infectious for 7 days (there is no latency period, see sub-model 6 below). Recrudescence can occur in the same individual only once within any given reproductive period. (d) Breeding occurs stochastically based on seasonally-varying, stage-specific probabilities. Breeding begins in late March (day-of-year 85) and lasts for two months (60 days) [8,9,10]. We assume all females that conceive, give birth. Females must be at least 24 months old to become pregnant and can conceive only once per year. At conception, the pregnancy counter of the female is initialized, and subsequently is advanced each day until the end of a 6-month (180-day) gestation period [8,9,10]. (e) Births occur at the end of a 26-week gestation period, with 80% of the mature females giving birth to 1 male or 1 female pup with equal probability, and 90% of the births occurring during October. Pups born to I or R mothers are born with maternal immunity (infection status = Mi) to Hendra [7,11] whereas pups born to S or E mothers are born susceptible (infection status = S) to Hendra. At birth, the pup dependency counter of the female is initialized, and subsequently is advanced each day until the end of a 5-month pup dependency period [8,9,10]. The pregnancy counter of the female is reset (to 99999) for the next breeding season. The pup dependency counter of the female also is monitored in this sub-model and is reset (to 99999) for the next breeding season when the period of pup dependency ends. (f) Disease transmission occurs stochastically with the daily probability (Pr) that a susceptible individual (S) becomes exposed and latently infected (E) calculated as the product of a contact rate (c = 19), a fixed transmission probability per encounter between S and I individuals (β = 4.76E-5), and the current number of S and I individuals in the population: Pr = (c * β * S * I) / N, where N represents population size [12]. The value of c was calibrated such that a population with 95% susceptible individuals at the beginning of an epidemic (at the appearance of the first infectious individual) would attain a maximum of 60% infectious individuals during the epidemic [5]. Upon infection, the infection status of the newly-infected individual is changed from S to E and its latency counter is initialized, and subsequently is advanced each day until the end of a 6-day latency period [13], at which time it is reset (to 99999) until subsequent infection. At the end of the latency period, the infection status of the individual is changed from E to I and its infectious counter is initialized, and subsequently is advanced each day until the end of a 7-day infectious period [13], at which time it is reset (to 99999) until the individual again changes infection status from E to I. The maternal immunity period counter also is updated in this sub-model and the infection status of individuals is changed as appropriate. The maternal immunity counter of each young-of-the-year (P and J) is advanced each day until the end of a 6-month maternal immunity period [13] and then is reset (to 99999) for life. Upon loss of maternal immunity, the infection status of the individual is changed from maternally immune (Mi) to susceptible (S). (g) System-level output produced. System-level output includes total population size, and numbers and proportions of flying-foxes in each age class, life-history stage, and infection status.

***References***

1. Grimm V, Berger U, Bastiansen F, Eliassen S, Ginot V, et al. (2006) A standard protocol for describing individual-based and agent-based models. Ecological Modelling 198: 115-126.

2. Rahman SA, Hassan SS, Olival KJ, Mohamed M, Chang L-Y, et al. (2010) Characterization of Nipah virus from naturally infected *Pteropus vampyrus* bats, Malaysia. Emerging Infectious Diseases 16: 1990-1993.

3. Field H, Kung N (2011) Henipaviruses - unanswered questions of lethal zoonoses. Current Opinion in Virology 1: 658-661.

4. Roberts BJ, Catterall CP, Eby P, Kanowski J (2012) Long-distance and frequent movements of the flying-fox *Pteropus poliocephalus*: implications for management. PLoS ONE 7: e42532. doi:42510.41371/journal.pone.0042532.

5. Field H (2005) The ecology of Hendra virus and Australian bat lyssavirus. Brisbane, Australia: The University of Queensland. 233 p.

6. Mcllwee AP, Martin L (2002) On the intrinsic capacity for increase of Australian flying-foxes (*Pteropus spp*., *Megachiroptera*) The Australian Zoologist 32: 76-100.

7. Breed AC, Breed MF, Meers J, Field HE (2011) Evidence of endemic Hendra virus infection in flying-foxes (*Pteropus conspicillatus*) - implications for disease risk management. PLoS ONE 6: e28816. doi:28810.21371/journal.pone.0028816.

8. Nelson J (1965) Movements of Australian flying foxes (Pteropodidae: Megachiroptera). Australian Journal of Zoology 13: 53-74.

9. Vardon MJ, Tidemann CR (1998) Reproduction, growth and maturity in the black flying-fox, *Pteropus alecto* (Megachiroptera : Pteropodidae). Australian Journal of Zoology 46: 329-344.

10. Martin SW, Meek AH, Willberg P (1987) Veterinary epidemiology - principles and methods. Ames, IA: Iowa State University Press.

11. Plowright RK, Field HE, Smith C, Divljan A, Palmer C, et al. (2008) Reproduction and nutritional stress are risk factors for Hendra virus infection in little red flying foxes (*Pteropus scapulatus*). Proceedings of the Royal Society B: Biological Sciences 275: 861-869.

12. Anderson RM, May RM (1991) Infectious diseases of humans: dynamics and control. Oxford, UK: Oxford Science Publications.

13. Plowright RK, Foley P, Field HE, Dobson AP, Foley JE, et al. (2011) Urban habituation, ecological connectivity and epidemic dampening: the emergence of Hendra virus from flying foxes (*Pteropus* spp.). Proceedings of the Royal Society B: Biological Sciences 278: 3703-3712.
